# Diagnostic and Prognostic Value of Ferritin and Soluble Interleukin-2 Receptor α in Critically Ill Children with Hyperferritinemia

**DOI:** 10.3390/diagnostics16162600

**Published:** 2026-08-17

**Authors:** İlknur Pençe, Hazal C. Tuğrul, Mahmud Esad Pençe, Gürkan Atay, Sibel Kuraş, Ceren Bilgün, Seher Erdoğan

**Affiliations:** 1 Department of Pediatrics, Göztepe Prof. Dr. Süleyman Yalçın City Hospital, Faculty of Medicine, Istanbul Medeniyet University, Istanbul 34722, Türkiye; 2 Department of Pediatric Intensive Care, Health Sciences University Ümraniye Training and Research Hospital, Istanbul 34764, Türkiye; hazaltugrul@gmail.com (H.C.T.); drgurkanatay@yahoo.com (G.A.); hcerent@hotmail.com (C.B.); seher70@gmail.com (S.E.); 3 Department of Medical Biochemistry, Faculty of Medicine, Marmara University, Istanbul 34854, Türkiye; esad.pence@marmara.edu.tr; 4 Department of Biochemistry, Faculty of Medicine, Health Sciences University, Istanbul 34668, Türkiye; sibel.kuras@sbu.edu.tr

**Keywords:** hemophagocytic lymphohistiocytosis, soluble interleukin-2 receptor, ferritin, macrophage activation syndrome, critically ill children, sepsis, mortality, ROC curve

## Abstract

**Background/Objectives:** Ferritin and soluble interleukin-2 receptor α (sIL-2Rα) are HLH-related biomarkers, but their comparative discrimination in pediatric critical illness is uncertain. We primarily compared their discrimination for adjudicated HLH in hyperferritinemic PICU children and secondarily assessed PICU mortality. **Methods:** This prospective single-center study enrolled 75 children aged 1 month–18 years with ferritin ≥500 ng/mL: 25 each with HLH, sepsis/septic shock, or other diseases. Both biomarkers were measured from the same admission sample. Research-use sIL-2Rα was reported in ng/mL without cross-calibration to the HLH-2004 U/mL scale; adjudicators were blinded to sIL-2Rα, whereas ferritin informed the reference process. Analyses used ROC, Firth regression, targeted post hoc paired AUC tests with Holm adjustment, bootstrap optimism correction, and cross-validation. **Results:** For adjudicated HLH, ferritin showed greater discrimination than sIL-2Rα (AUC, 0.898 vs. 0.690; ΔAUC, 0.208; Holm-adjusted *p* = 0.019). The exploratory 1614-ng/mL ferritin cutoff had 92% sensitivity and 76% specificity. Although sIL-2Rα remained associated with HLH after ferritin adjustment (OR, 1.089 per ng/mL; *p* = 0.002), adding it did not significantly improve discrimination beyond ferritin (*p* = 0.663). For secondary mortality, ferritin showed greater discrimination than sIL-2Rα (AUC, 0.754 vs. 0.495; Holm-adjusted *p* = 0.019). Adding ferritin to PRISM III increased the apparent AUC from 0.725 to 0.811, but the comparison was not statistically significant (*p* = 0.105); the bootstrap optimism-corrected AUC was 0.800. **Conclusions:** In this hyperferritinemic PICU cohort, ferritin showed greater discrimination for adjudicated HLH than the studied sIL-2Rα assay, and adding sIL-2Rα did not significantly improve discrimination. Findings should be interpreted in the context of the studied assay and reference process. The exploratory mortality model requires external validation.

## 1. Introduction

Hyperferritinemia is frequently encountered in pediatric critical illness and may reflect several overlapping processes, including sepsis, hemophagocytic lymphohistiocytosis (HLH), macrophage activation syndrome (MAS), and tissue injury [1,2]. Each year, approximately 239,000 children are admitted to pediatric intensive care units (PICUs) in the United States [3]. PICU mortality varies across patient populations and resource settings [4,5,6]. Identifying biomarkers that distinguish patients at highest risk for poor outcomes is therefore important for risk stratification and treatment selection.

Ferritin, an acute-phase reactant reflecting macrophage activation, is elevated across a spectrum of pediatric critical illnesses, including sepsis, HLH, and MAS [1,2]. Distinguishing HLH from sepsis is clinically important [7,8] because these conditions often require different treatment priorities, including HLH-directed immunomodulation versus source control, antimicrobial therapy, and supportive care in sepsis. Extremely elevated ferritin levels may help identify HLH/MAS patients requiring early immunosuppression [9], and hyperferritinemia has been associated with increased mortality in pediatric and adult critical illness [1,2,10].

Soluble interleukin-2 receptor α (sIL-2Rα), shed by activated T cells, is a key diagnostic criterion in the HLH-2004 guidelines [7,11]. Elevated sIL-2Rα reflects T-cell activation and has been proposed as an early diagnostic marker, as levels may rise before classic HLH manifestations appear [8,12]. However, the diagnostic value of sIL-2Rα relative to ferritin in the PICU setting remains unclear.

Despite their individual utility, the diagnostic and prognostic performance of ferritin and sIL-2Rα, evaluated in the same cohort individually and in combination, has been insufficiently studied in pediatric critical illness. Because misclassification carries direct therapeutic consequences, clarifying how these biomarkers discriminate HLH from its mimics has immediate clinical relevance. We therefore compared these biomarkers, individually and in combination, in a cohort of critically ill children with hyperferritinemia, evaluating their utility for HLH diagnosis, their prognostic value for PICU mortality, and their correlations with markers of organ injury.

## 2. Materials and Methods

### 2.1. Study Design and Patients

**Figure 1:**
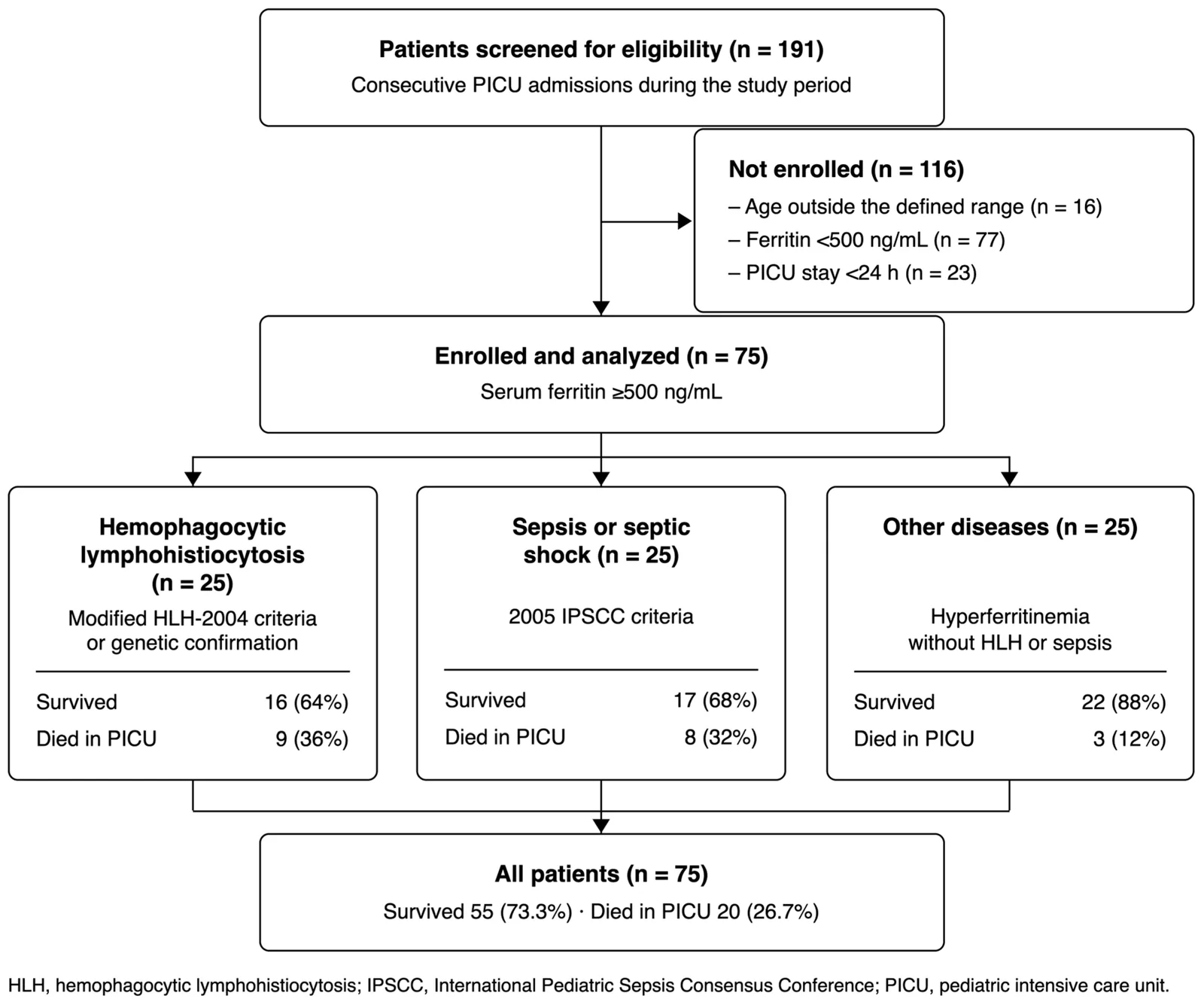
Participant screening, enrollment, diagnostic-group classification, and PICU mortality. Of 191 consecutive PICU admissions in whom ferritin was measured, 116 were not enrolled because of age outside the defined range (*n* = 16), ferritin < 500 ng/mL (*n* = 77), or PICU stay < 24 h (*n* = 23), leaving 75 enrolled patients classified into three diagnostic groups (25 per group). Twenty patients (26.7%) died during PICU stay.

This single-center, prospective observational study was conducted in a 15-bed, Level 3 Pediatric Intensive Care Unit (PICU) from 1 July 2023 to 1 May 2024. The study was conducted in accordance with the Declaration of Helsinki and approved by the Ümraniye Training and Research Hospital Ethics Committee (protocol code 254, approved 16 June 2023). Written informed consent was obtained from the parents or legal guardians of all participants. During the study period, 191 consecutive PICU admissions in whom serum ferritin was measured were screened. Patients were eligible if they were aged 1 month to 18 years, had a ferritin concentration ≥500 ng/mL, and remained in the PICU for at least 24 h. The mutually exclusive reasons for non-enrollment were age outside the defined range (*n* = 16), ferritin < 500 ng/mL (*n* = 77), and PICU stay < 24 h (*n* = 23), leaving 75 patients who were enrolled and analyzed (Figure 1). The primary objective was to evaluate the ability of ferritin and sIL-2Rα to discriminate HLH from non-HLH conditions; the secondary objective was to assess their prognostic value for PICU mortality. To achieve balanced diagnostic groups, eligible patients were enrolled consecutively within each category until each prespecified group reached 25 patients, yielding Sepsis/Septic Shock (*n* = 25), Primary/Secondary HLH (*n* = 25), and Other Diseases (*n* = 25); no eligible patient was excluded because a group was already complete.

Sepsis and septic shock were classified according to the 2005 International Pediatric Sepsis Consensus Conference (IPSCC) criteria [13]. Sepsis was defined as systemic inflammatory response syndrome in the presence of, or resulting from, suspected or proven infection. Septic shock was defined as sepsis with cardiovascular organ dysfunction. After administration of ≥40 mL/kg of isotonic intravenous fluid within 1 h, cardiovascular dysfunction was identified by age-specific hypotension, vasoactive support at IPSCC-specified thresholds, or at least two of the following: unexplained metabolic acidosis with a base deficit >5 mEq/L, arterial lactate >2 times the upper limit of normal, urine output <0.5 mL/kg/h, capillary refill >5 s, or a core-to-peripheral temperature gap >3 °C. HLH was diagnosed based on the modified HLH-2004 diagnostic criteria [7] or through molecular/genetic confirmation. Because NK-cell activity and sIL-2Rα (sCD25) levels were not routinely available at our center, patients were evaluated using the remaining six HLH-2004 criteria: fever (≥38.5 °C), splenomegaly, cytopenias affecting at least two lineages, hypertriglyceridemia and/or hypofibrinogenemia, hemophagocytosis in bone marrow or tissue, and hyperferritinemia (≥500 ng/mL). Patients meeting at least five of the six assessable criteria or those with a confirmed genetic mutation were classified as HLH. Because ferritin ≥500 ng/mL was also an eligibility criterion, this item was satisfied by definition in all enrolled participants; in the absence of genetic confirmation, classification therefore required at least four of the five remaining assessable criteria.

The pediatric intensivist (S.E.), in consultation with pediatric hematology, adjudicated whether pathologic immune activation was the most likely underlying process; patients whose findings were fully explained by alternative pathologies, such as isolated liver failure, were excluded from the HLH group. The “Other Diseases” group comprised PICU patients with ferritin ≥500 ng/mL who fulfilled neither the modified HLH-2004 nor the IPSCC sepsis criteria; patients were classified by the primary underlying condition necessitating PICU admission: malignancy (hematologic or solid tumor; *n* = 7; 28.0%); cardiovascular disorders (*n* = 4; 16.0%); metabolic diseases (*n* = 4; 16.0%); bone-marrow or solid-organ transplantation (*n* = 2; 8.0%); cerebral palsy, with or without comorbidities (*n* = 2; 8.0%); other neurological sequelae, including post-meningitis sequelae (*n* = 2; 8.0%); post-operative critical illness (*n* = 1; 4.0%); primary immunodeficiency (*n* = 1; 4.0%); post-traumatic critical illness (*n* = 1; 4.0%); and epilepsy (*n* = 1; 4.0%) (Supplementary Table S6). In patients with hematologic malignancy, HLH classification was based on overall clinicopathologic adjudication rather than bone-marrow hemophagocytosis alone; the absence of hemophagocytosis was not considered sufficient by itself to exclude HLH when other features supported pathologic immune activation. Diagnostic adjudication was conducted blind to sIL-2Rα results, which were batch-analyzed separately (Section 2.4) and were not available during clinical decision-making; ferritin, as a component of the HLH-2004 criteria, was necessarily available to the adjudicators and was therefore not masked. The ferritin criterion was applied dichotomously at the HLH-2004 threshold of ≥500 ng/mL; ferritin concentrations above this threshold received no additional weight in the formal classification rule. Patients were assigned to mutually exclusive diagnostic groups based on the adjudicated primary process at PICU admission, when the study sample was obtained. Sepsis developing later during the PICU stay did not lead to reclassification.

### 2.2. Data Collection

Upon PICU admission, blood samples were drawn for routine laboratory assessments, including complete blood count, biochemical profiles, coagulation parameters, blood gas analysis, C-reactive protein (CRP), and procalcitonin. Ferritin and sIL-2Rα were measured in the same sample obtained at the first blood draw after PICU admission. Medication records were reviewed for treatment administered before the index sample; none of the 75 enrolled patients had received corticosteroid therapy before sampling. Serum was separated by centrifugation (3000× *g*, 10 min, room temperature) for sIL-2Rα measurement and stored at −80 °C until batch analysis. Clinical and demographic data were collected from medical records, including Pediatric Risk of Mortality III (PRISM III) and Pediatric Logistic Organ Dysfunction (PELOD) scores calculated using data from the first 24 h of PICU admission.

### 2.3. Ferritin Measurement

Serum ferritin was measured via electrochemiluminescence immunoassay (ECLIA) on a cobas e601 analyzer (Roche Diagnostics, Basel, Switzerland). The analytical range was 0.50–2000 ng/mL; samples exceeding 2000 ng/mL were automatically diluted (1:50), allowing quantification up to 100,000 ng/mL.

### 2.4. sIL-2Rα Measurement

Serum sIL-2Rα (sCD25) levels were measured using a commercial human sCD25 ELISA kit (CK-bio-13405, Coon Koon Bio, Shanghai, China) according to the manufacturer’s instructions (sensitivity: 0.1 ng/mL; manufacturer-reported intra-assay coefficient of variation (CV) <8%, inter-assay CV <10%). Samples were diluted 1:5 prior to the assay; final concentrations were back-calculated (×5), extending the effective range to 5–200 ng/mL. sIL-2Rα was measured solely as a research biomarker and was not used to establish the diagnosis of HLH.

### 2.5. Statistical Analysis

The required sample size was calculated a priori in G*Power 3.1.9.7 for an omnibus one-way ANOVA across three groups (α = 0.05, 80% power, Cohen’s f = 0.40), yielding a minimum of 66 participants; the target of 75 corresponded to 86.9% nominal power under these assumptions. Because the calculation addressed between-group biomarker differences rather than diagnostic-accuracy precision or multivariable performance, cohort-derived cutoffs and model estimates were considered exploratory.

Normality was assessed using the Shapiro–Wilk and Kolmogorov–Smirnov tests. Continuous variables are summarized as mean ± SD or median (IQR) and were compared using Student’s *t*-test, Mann–Whitney U test, one-way ANOVA, or Kruskal–Wallis test, as appropriate; significant Kruskal–Wallis tests were followed by Dunn’s tests with Benjamini–Hochberg adjustment. Spearman correlation was used for non-normally distributed variables. Categorical variables were compared using χ^2^ or Fisher’s exact tests, and between-group differences in correlations using Fisher’s r-to-z transformation (Supplementary Table S1).

Diagnostic and biomarker-only mortality models included log_10_-transformed ferritin and untransformed sIL-2Rα. Given the small sample and sparse outcomes (*n* = 75; 25 HLH cases; 20 deaths), all logistic models used Firth’s penalized maximum likelihood [14,15]; odds ratios are reported with profile penalized-likelihood 95% CIs and penalized likelihood-ratio *p* values. Discrimination was assessed by ROC analysis with Youden’s J statistic, and effect sizes by Cohen’s d with 95% CIs. Mortality modeling began with an extended Firth model including PRISM III, log_10_-transformed ferritin, and untransformed sIL-2Rα. Given 20 deaths and three candidate predictor parameters, model development was information-limited. Because sIL-2Rα was not associated with mortality and did not improve internally validated performance, a post hoc reduced candidate model retaining PRISM III and log_10_-transformed ferritin was reported; both specifications were internally validated (Supplementary Tables S3, S8 and S12).

AUCs were compared using paired DeLong tests. The two targeted post hoc standalone ferritin-versus-sIL-2Rα comparisons—adjudicated HLH and PICU mortality—were Holm-adjusted as one family (Supplementary Table S10). Incremental prognostic value beyond PRISM III was evaluated post hoc using paired bootstrap ΔAUC, repeated stratified cross-validated ΔAUC, and penalized likelihood-ratio testing (Supplementary Table S11). Full resampling, cross-validation, calibration, and model-comparison procedures are provided in the Supplementary Methods and Tables S3 and S8–S12. Apart from the Benjamini–Hochberg and Holm procedures specified above, no further multiplicity adjustment was applied to exploratory secondary analyses. No imputation was required. Analyses used IBM SPSS Statistics 25, Python 3.10 (scikit-learn 1.3), and R 4.6.0 (R Foundation for Statistical Computing, Vienna, Austria; logistf package version 1.26.1). All tests were two-sided with *p* < 0.05.

## 3. Results

Of 191 consecutive PICU admissions in whom ferritin was measured, 116 were not enrolled because of age outside the defined range (*n* = 16), ferritin < 500 ng/mL (*n* = 77), or PICU stay < 24 h (*n* = 23); 75 were enrolled and analyzed (Figure 1). Of the 75 participants, 39 (52%) were male, with ages ranging from 1 to 213 months. No significant differences were observed among groups in sex, age, body weight, PICU length of stay, or total hospital stay (Table 1).

### 3.1. Between-Group Comparisons

Ferritin and sIL-2Rα, the primary biomarkers of interest, both differed across the three groups (Table 2). Median ferritin was significantly higher in the HLH group (5239 ng/mL [IQR 2000–35,149]) than in sepsis (826 [582–1654]) and other diseases (978 [740–1413]; *p* < 0.001), with no difference between sepsis and other diseases (*p* = 0.80) (Supplementary Figure S2a). sIL-2Rα was also higher in HLH (83.8 ng/mL [70.9–90.0]) than in sepsis (74.9 [67.0–78.5]; *p* = 0.029, Dunn’s test) and other diseases (71.5 [60.3–82.5]; *p* = 0.015), with no difference between sepsis and other diseases (*p* = 0.861) (Supplementary Figure S2b).

Beyond the primary biomarkers, several supporting laboratory parameters also differed significantly across the three groups (Table 2): albumin (*p* = 0.003) and calcium (*p* = 0.008) were lowest in the HLH group (2.5 g/dL and 8.2 mg/dL), whereas AST (*p* < 0.001), ALT (*p* = 0.005), and LDH (*p* = 0.002) were highest in HLH (88, 42, and 751 U/L), and aPTT was longest in HLH (33.15 s; *p* = 0.023). CRP also differed across groups (*p* = 0.006), with HLH intermediate (38 mg/L) between sepsis (73 mg/L) and other diseases (8.2 mg/L). Effect sizes between HLH and the other groups were large to very large for ferritin and LDH and smaller for sIL-2Rα (for log_10_-transformed ferritin, Cohen’s d = 1.16 [95% CI 0.56–1.75] vs. sepsis and 1.09 [0.50–1.68] vs. other diseases; LDH d = 1.29 and 1.24; sIL-2Rα d = 0.76 and 0.80), with no substantial difference between sepsis and other diseases (ferritin d = −0.26; LDH d = −0.01) (Supplementary Table S7).

### 3.2. Diagnostic Performance of Ferritin and sIL-2Rα for HLH

**Figure 2:**
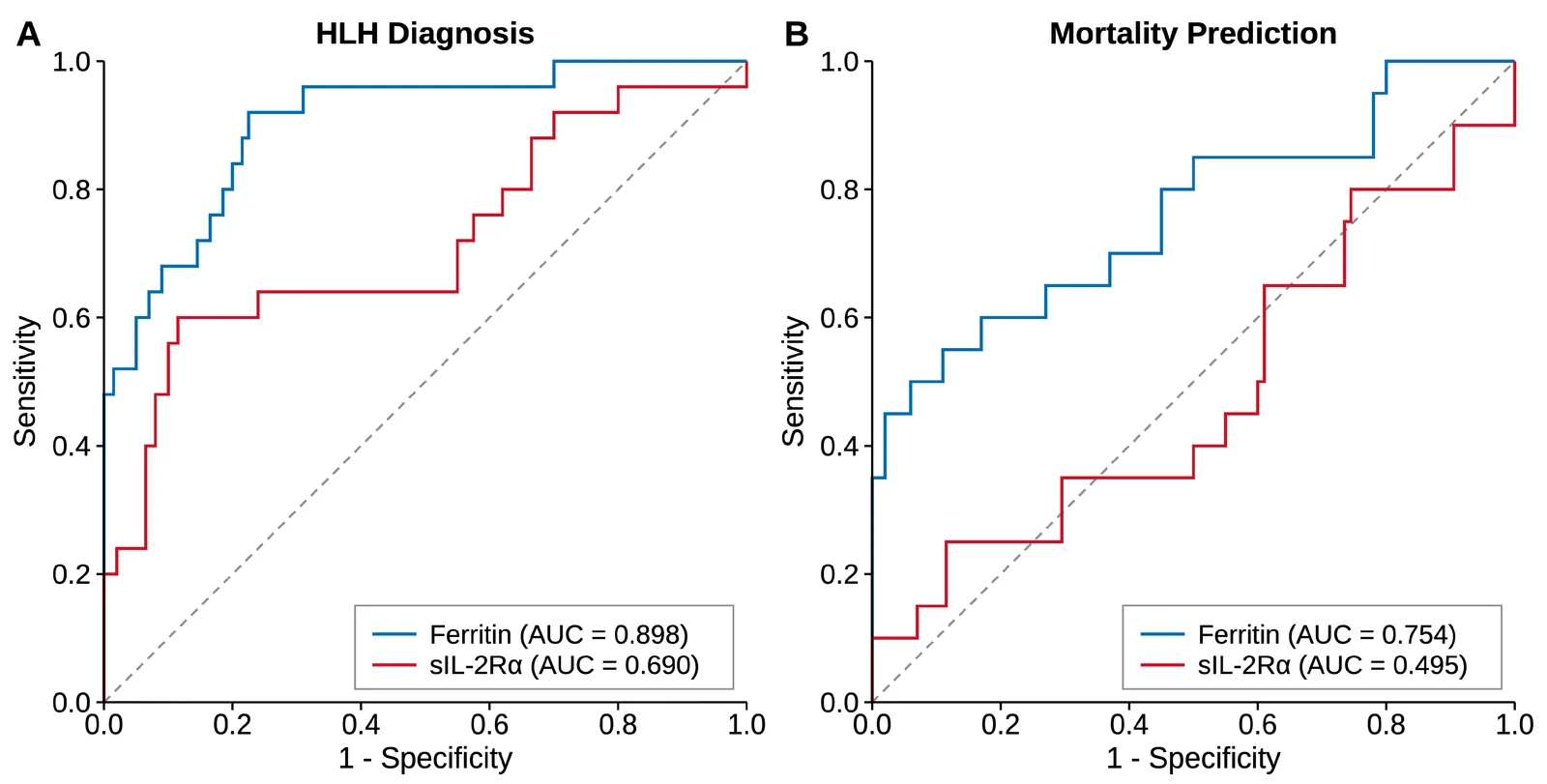
Receiver-operating-characteristic (ROC) curves for ferritin (blue) and soluble interleukin-2 receptor α (sIL-2Rα; red) in 75 critically ill children with hyperferritinemia. (**A**) HLH diagnosis (25 HLH vs. 50 non-HLH); (**B**) PICU mortality (20 non-survivors vs. 55 survivors). AUCs are shown in the figure; 95% CIs are given in Table 3. The dashed diagonal is the line of no discrimination. AUCs were compared using the paired DeLong test with Holm adjustment across the two comparisons: (**A**) ΔAUC 0.208, Holm-adjusted *p* = 0.019; (**B**) ΔAUC 0.260, Holm-adjusted *p* = 0.019 (Supplementary Table S10).

For HLH diagnosis, ferritin yielded an AUC of 0.898 (95% CI 0.818–0.965; sensitivity 92.0%, specificity 76.0% at a 1614 ng/mL cutoff), whereas sIL-2Rα yielded an AUC of 0.690 (0.548–0.817; sensitivity 60%, specificity 84% at 83.0 ng/mL) (Figure 2). In a targeted post hoc paired comparison of the standalone markers, ferritin had a higher AUC than sIL-2Rα for adjudicated HLH (ΔAUC, 0.208; 95% CI, 0.036–0.379; *p* = 0.018; Holm-adjusted *p* = 0.019) (Supplementary Table S10). A ferritin–sIL-2Rα diagnostic model had an apparent AUC of 0.933 (95% CI 0.867–0.979) with sensitivity 88.0% and specificity 84.0% (Supplementary Table S5), significantly outperforming sIL-2Rα alone (ΔAUC +0.242; 95% CI 0.035–0.449; *p* = 0.022) but not ferritin alone (ΔAUC +0.034; 95% CI, −0.119 to 0.187; *p* = 0.663) (Table 3, Panel A; Supplementary Table S4; model coefficients in Table 4, Panel A).

### 3.3. Mortality and Prognostic Models

Overall mortality was 26.7% (20/75): HLH 36% (9/25), sepsis 32% (8/25), and other diseases 12% (3/25). Non-survivors had higher PRISM III scores (median 22.5 vs. 18.0; *p* = 0.003), PELOD scores (median 31.5 vs. 21.0; *p* = 0.008), ferritin levels (median 4730.5 vs. 1007 ng/mL; *p* < 0.001), urea (median 35.5 vs. 23.0 mg/dL; *p* = 0.018), and aPTT (median 32.5 vs. 29.0 s; *p* = 0.025) compared with survivors (*n* = 55). No significant differences were observed for other parameters including sIL-2Rα (Table 5). Effect sizes were very large for PELOD (d = 1.67; 95% CI: 1.09–2.25), large for PRISM III (d = 0.95; 0.42–1.49) and ferritin (d = 0.80; 0.27–1.33).

For mortality prediction, ferritin yielded an AUC of 0.754 (95% CI, 0.620–0.881; cohort-derived cutoff, 1728 ng/mL), whereas sIL-2Rα yielded an AUC of 0.495 (95% CI, 0.345–0.651) (Figure 2B). In the targeted post hoc paired comparison, ferritin had the higher AUC (ΔAUC, 0.260; 95% CI, 0.064–0.455; Holm-adjusted *p* = 0.019; Supplementary Table S10). The parsimonious ferritin–PRISM III model (log_10_-transformed ferritin: OR, 5.51; *p* < 0.001; PRISM III: OR, 1.089; *p* = 0.019) had an apparent AUC of 0.811 and a bootstrap optimism-corrected AUC of 0.800 (Table 3 and Table 4). PRISM III alone yielded an AUC of 0.725, and the difference was not statistically significant by DeLong’s test (ΔAUC, 0.087; 95% CI, −0.018 to 0.192; *p* = 0.105). Its bootstrap optimism-corrected AUC was 0.723; bootstrap optimism-corrected Brier scores were 0.138 and 0.183 and calibration slopes 1.02 and 1.20 for the ferritin–PRISM III and PRISM III-only models, respectively (Supplementary Table S12). Paired bootstrap gave a ΔAUC of 0.087 (95% percentile interval, 0.005–0.189) and repeated 5-fold cross-validation a mean out-of-fold ΔAUC of 0.083 (2.5th–97.5th percentile, 0.055–0.114; Supplementary Table S11). The Firth penalized likelihood-ratio test for adding ferritin to the PRISM III model yielded *p* = 1.5 × 10^−4^ (Table 4, Panel B). Because these estimates derive from the same 20 deaths, they provide complementary internal support rather than independent confirmation. Adding sIL-2Rα did not improve prognostic performance.

### 3.4. Correlation of Ferritin and sIL-2Rα with Clinical Parameters

Across the full cohort (*n* = 75), 48 exploratory Spearman correlation tests were performed without multiplicity adjustment; the *p* values reported below are nominal. Ferritin showed positive correlations with AST (ρ = 0.397; *p* < 0.001), ALT (ρ = 0.357; *p* = 0.002), LDH (ρ = 0.318; *p* = 0.006), urea (ρ = 0.313; *p* = 0.006), and aPTT (ρ = 0.244; *p* = 0.035), whereas nominal *p* values did not fall below 0.05 for Na, PELOD, HCO_3_, Ca, CRP, or white blood cell count. sIL-2Rα showed a weak negative correlation with INR (ρ = −0.240; *p* = 0.038) and no other nominal *p* values fell below 0.05, including with triglycerides (ρ = 0.177; *p* = 0.129) and ALT (ρ = 0.076; *p* = 0.518) (Supplementary Figure S1). In diagnosis-stratified analyses, the ferritin–AST correlation did not reach nominal *p* < 0.05 in any subgroup (HLH ρ = 0.273, *p* = 0.186; sepsis ρ = 0.199, *p* = 0.341; other diseases ρ = 0.134, *p* = 0.523), and no between-group differences in sIL-2Rα correlation coefficients were detected.

## 4. Discussion

In this prospective, balanced cohort of 75 critically ill children with hyperferritinemia, ferritin discriminated adjudicated HLH with an AUC of 0.898, compared with 0.690 for sIL-2Rα, and a combined ferritin–sIL-2Rα model had an apparent AUC of 0.933. sIL-2Rα remained associated with HLH after adjustment for ferritin, but the combined model did not significantly exceed ferritin alone. For PICU mortality, ferritin, but not sIL-2Rα, was associated with outcome. The ferritin–PRISM III model had a bootstrap optimism-corrected AUC of 0.800. Its apparent AUC increment over PRISM III alone was not statistically significant by DeLong testing; complementary post hoc analyses yielded positive within-cohort findings, but external validation is required before clinical use. Because ferritin informed the diagnostic reference process, the diagnostic comparison reflects within-cohort discrimination rather than assay-independent superiority.

Prior studies in adults have compared these biomarkers with differing conclusions; Hayden et al. reported a higher observed AUC for sIL-2R than for ferritin (0.90 vs. 0.78) among adults evaluated for suspected HLH [16]. Previous studies have reported ferritin cutoffs for HLH ranging from approximately 1700 to >10,000 ng/mL, reflecting differences in patient populations and disease spectra [9,17,18,19]. Our exploratory cutoff of 1614 ng/mL lies just below the lower bound of this range and requires external validation.

Prior studies of combined ferritin and sIL-2Rα for HLH diagnosis have also yielded conflicting results: the Optimized HLH Inflammatory Index achieved AUC 0.87 in adult malignancy-associated HLH [20], whereas Hayden et al. found no improvement over sIL-2R alone [16]. In our cohort, the ferritin–sIL-2Rα diagnostic model had an apparent AUC of 0.933 but did not significantly outperform ferritin alone (ΔAUC +0.034; *p* = 0.663), although it significantly exceeded sIL-2Rα alone (ΔAUC +0.242; *p* = 0.022) (Table 3, Panel A; Supplementary Table S4). The discrepancy with Hayden’s single-marker AUC ranking may reflect differences in age group, case mix, and assay platform; that retrospective series used a clinical assay reporting sIL-2R in U/mL, whereas our research-use assay reported in ng/mL without cross-calibration. The studies also differed in their reference processes: in Hayden et al., both ferritin and sCD25 could contribute to the HLH-2004-based classification against which they were evaluated, whereas here, within the formal criterion count, ferritin contributed as a dichotomous item positive in every participant and the research-use sIL-2Rα result was masked and excluded. These asymmetric reference-process dependencies limit direct comparison of the observed single-marker AUC rankings.

The HLH-2004 guidelines include sIL-2Rα ≥2400 U/mL as one diagnostic criterion, but clinical interpretation is limited by the lack of international assay standardization [11,21]. Our ELISA reports in ng/mL, precluding direct application of the U/mL threshold; we derived a cohort-specific cutoff of 83.0 ng/mL (sensitivity 60%, specificity 84%, AUC 0.690), underscoring the need for platform-specific validation [22,23].

In the pooled cohort, ferritin co-varied with AST, ALT, and LDH, biochemical markers of hepatic and tissue injury [24], whereas no comparable pattern was detected for sIL-2Rα. These unadjusted exploratory associations may partly reflect between-group case mix and do not establish directionality, causality, or a specific biological mechanism. HLH patients had lower CRP than those with sepsis despite higher ferritin, consistent with prior reports [25,26,27]; however, CRP was not a primary endpoint, and this observation remains exploratory.

Both PRISM III and PELOD scores were higher in non-survivors [28,29,30]. In the exploratory multivariable analysis, ferritin remained associated with PICU mortality after adjustment for PRISM III (OR 5.51; *p* < 0.001), whereas sIL-2Rα was not associated with mortality (*p* = 0.81, biomarker-only mortality model; Supplementary Table S2). This finding is consistent with Fan et al. [31], who reported an OR of 4.85 (95% CI, 2.55–9.60) for mortality in children with hyperferritinemic sepsis. Adding sIL-2Rα did not improve prognostic performance, and its bootstrap coefficient interval included zero (Supplementary Table S3). Standard DeLong inference may be conservative for nested models fitted and evaluated in the same cohort [32]; therefore, the DeLong result was interpreted alongside complementary model-based and resampling analyses. The Firth penalized likelihood-ratio test assessed the contribution of ferritin conditional on PRISM III, paired bootstrap quantified uncertainty in the AUC increment, and repeated cross-validation examined the stability of the increment in out-of-fold predictions. Because all analyses were based on the same 20 deaths, they provide complementary internal evidence rather than independent validation. These post hoc analyses do not establish incremental prognostic performance. The findings therefore justify external validation of the parsimonious model, not clinical use. Previous studies reported higher mortality cutoffs in disease-specific cohorts (2375 ng/mL in sepsis [33]; 2161 ng/mL in HLH [34]); our lower cutoff of 1728 ng/mL likely reflects diagnostic heterogeneity.

The single-center setting and eligibility criteria based on age, ferritin ≥500 ng/mL, and PICU stay ≥24 h restricted the case mix and transportability, particularly because the ferritin threshold narrowed the biomarker and disease spectrum. The balanced 25/25/25 design did not reflect natural prevalence, so prevalence-dependent predictive values were not estimated. Ferritin was one of six assessable criteria and visible to adjudicators but contributed to formal classification only as a dichotomous item at ≥500 ng/mL. Because this threshold was also required for eligibility, the criterion was positive in every participant and therefore had no between-participant variation; concentrations above the threshold received no additional weight. Thus, the reference process was not fully independent of ferritin; conversely, the research-use sIL-2Rα result was masked and excluded from classification, and clinical sCD25 and NK-cell activity were unavailable. The restricted case mix may produce spectrum or selection effects, and the reference-process structure introduces potential incorporation bias; the magnitude and direction of their combined effect on observed diagnostic estimates cannot be determined. The paired diagnostic AUC comparison therefore reflects within-cohort discrimination rather than assay-independent superiority and should not be generalized beyond this selected hyperferritinemic cohort without external validation. Written informed consent and ethics approval covered enrolled participants only, so patient-level data from non-enrolled admissions could not be analyzed; the screening cascade is reported in aggregate (Figure 1).

The research-use sIL-2Rα ELISA reported ng/mL rather than the U/mL scale used for the HLH-2004 sCD25 criterion; because no analytical bridge was established, its concentrations and thresholds are not directly comparable with that criterion. Its analytical performance was not compared with clinically validated platforms, so the observed discrimination is assay-specific and should not be assumed to generalize to those platforms. The mortality models were based on 20 events, and secondary and subgroup analyses (*n* = 25 per group) are hypothesis-generating. Both biomarkers were measured once at PICU admission, and the interval from symptom onset to admission was not recorded; this design cannot account for between-patient disease-stage heterogeneity or longitudinal biomarker changes.

Strengths include the prospective design, contemporaneous measurement of both biomarkers from the same admission sample, masking of sIL-2Rα during diagnostic adjudication, paired comparison of the standalone biomarkers, Firth penalized regression, and complementary bootstrap and cross-validation procedures.

## 5. Conclusions

In this balanced single-center hyperferritinemic PICU cohort, ferritin showed greater observed discrimination for adjudicated HLH than the research-use sIL-2Rα assay, although the comparison was conditioned on a ferritin-informed reference process; adding sIL-2Rα did not significantly improve discrimination. The assay’s ng/mL results were not cross-calibrated to the HLH-2004 sCD25 U/mL criterion, and its observed discrimination is platform-specific. Ferritin remained associated with PICU mortality after adjustment for PRISM III, but the exploratory ferritin–PRISM III model requires external validation before clinical use.

## Figures and Tables

**Table 1:** Comparison of demographic and clinical characteristics between patient groups.

Characteristic	HLH (*n* = 25)	Sepsis (*n* = 25)	Other Diseases (*n* = 25)	*p* Value
Age, months	51 (12–101)	49 (4–134)	58 (22–88)	0.857
Weight, kg	16 (9.5–27)	16 (6–36)	18 (11.5–24)	0.849
Systolic BP, mmHg ^†^	95 ± 23.7	90 ± 22.3	102 ± 25.0	0.205
Diastolic BP, mmHg ^†^	63 ± 18.0	69 ± 16.9	70 ± 25.0	0.423
PICU stay, days	14 (5–30)	14 (8–26)	9 (3–37)	0.651
Hospital stay, days	19 (14–45)	29 (14–45)	34 (11–57)	0.957
Sex, *n* (%)				0.146
Female	16 (64)	10 (40)	10 (40)	
Male	9 (36)	15 (60)	15 (60)	

Data are median (IQR) or *n* (%) unless otherwise indicated. *p* values were calculated using the Kruskal–Wallis or χ^2^ test, as appropriate. ^†^ Blood-pressure data, available from separate records, are presented as mean ± SD and were compared using one-way ANOVA. BP, blood pressure; HLH, hemophagocytic lymphohistiocytosis; IQR, interquartile range; PICU, pediatric intensive care unit.

**Table 2:** Comparison of laboratory parameters between patient groups.

Parameter	HLH (*n* = 25)	Sepsis (*n* = 25)	Other Diseases (*n* = 25)	*p* Value
Ferritin, ng/mL	5239 (2000–35,149)	826 (582–1654)	978 (740–1413)	**<0.001**
sIL-2Rα, ng/mL	83.8 (70.9–90.0)	74.9 (67.0–78.5)	71.5 (60.3–82.5)	**0.028**
WBC, ×10^3^/μL	9.96 (3.78–17.17)	12.10 (6.87–20.18)	9.68 (7.84–14.17)	0.308
Neutrophils, ×10^3^/μL	6.59 (1.94–12.12)	6.29 (3.79–11.58)	7.05 (4.39–8.84)	0.836
Lymphocytes, ×10^3^/μL	1.40 (0.71–4.40)	2.47 (1.50–4.55)	1.35 (1.03–2.68)	0.123
Hemoglobin, g/dL	9.0 (8.5–10.2)	10.9 (9.5–11.6)	10.4 (8.7–11.4)	0.147
Hematocrit, %	28.4 (26.6–31.5)	31.7 (29.5–35.2)	32.0 (26.5–34.0)	0.154
Platelets, ×10^3^/μL	126 (27–279)	172 (109–371)	157 (91–335)	0.181
AST, U/L	88 (49–320)	37 (23–49)	52 (23–110)	**<0.001**
ALT, U/L	42 (28–116)	19 (9–31)	37 (15–176)	**0.005**
Creatinine, mg/dL	0.39 (0.24–1.00)	0.23 (0.17–0.45)	0.35 (0.23–0.63)	0.099
Albumin, g/dL	2.5 (2.5–3.25)	3.3 (2.8–3.9)	3.7 (3.1–4.2)	**0.003**
Fibrinogen, mg/dL	154 (89–307)	278 (179–364)	215 (128–311)	0.267
Sodium, mmol/L	138 (134–141)	137 (135–139)	139 (138–141)	0.121
Calcium, mg/dL	8.2 (7.6–8.4)	8.9 (8.1–9.1)	8.4 (8.0–9.1)	**0.008**
CRP, mg/L	38 (8.8–99)	73 (7.1–127)	8.2 (2.4–27)	**0.006**
Procalcitonin, ng/mL	2.95 (0.40–20.4)	0.35 (0.08–5.3)	0.30 (0.06–6.9)	0.120
Triglycerides, mg/dL	238.5 (143–489)	167.5 (155–237)	204.5 (117–389)	0.776
LDH, U/L	751 (518–1236)	352 (271–551)	353 (275–598)	**0.002**
INR	1.28 (1.10–1.49)	1.23 (1.13–1.42)	1.30 (1.03–1.59)	0.902
aPTT, s	33.15 (29.0–37.9)	29.0 (26.0–33.1)	27.3 (24.0–33.0)	**0.023**
Lactate, mmol/L	2.29 (1.54–4.53)	2.20 (1.40–3.10)	1.78 (1.28–2.04)	0.088
Bicarbonate, mmol/L	20.8 (17.0–23.0)	21.3 (20.0–23.0)	22.25 (20.45–24.25)	0.287

Data are median (IQR). Bold *p* values indicate statistical significance (*p* < 0.05); *p* values were calculated using the Kruskal–Wallis test. ALT, alanine aminotransferase; aPTT, activated partial thromboplastin time; AST, aspartate aminotransferase; CRP, C-reactive protein; HLH, hemophagocytic lymphohistiocytosis; INR, international normalized ratio; IQR, interquartile range; LDH, lactate dehydrogenase; sIL-2Rα, soluble interleukin-2 receptor α; WBC, white-blood-cell count.

**Table 3:** Diagnostic and prognostic discrimination of ferritin and sIL-2Rα.

Model (Cutoff)	AUC (95% CI)	Sensitivity, % (95% CI)	Specificity, % (95% CI)	LR+ (95% CI)	LR− (95% CI)
**A|HLH diagnosis**					
Ferritin (≥1614 ng/mL)	0.898 (0.818–0.965)	92.0 (75.0–97.8)	76.0 (62.6–85.7)	3.83 (2.31–6.36)	0.11 (0.03–0.40)
sIL-2Rα (≥83.0 ng/mL)	0.690 (0.548–0.817)	60.0 (40.7–76.6)	84.0 (71.5–91.7)	3.75 (1.84–7.64)	0.48 (0.29–0.78)
Combined model ^a^ (*p* ≥ 0.34 ^b^)	0.933 (0.867–0.979)	88.0 (70.0–95.8)	84.0 (71.5–91.7)	5.50 (2.87–10.55)	0.14 (0.05–0.42)
**B|PICU mortality**					
Ferritin (≥1728 ng/mL)	0.754 (0.620–0.881)	65.0 (43.3–81.9)	69.1 (56.0–79.7)	2.10 (1.26–3.50)	0.51 (0.27–0.94)
sIL-2Rα	0.495 (0.345–0.651)	—	—	—	—
PRISM III alone	0.725 (0.593–0.857)	—	—	—	—
Ferritin + sIL-2Rα (*p* ≥ 0.55 ^b^)	0.753 (0.614–0.880)	45.0 (25.8–65.8)	98.2 (90.4–99.7)	24.75 (3.34–183.18)	0.56 (0.38–0.83)
Parsimonious ferritin + PRISM III model ^c^ (*p* ≥ 0.33 ^b^)	0.811 ^d^	70.0 (48.1–85.5)	90.9 (80.4–96.1)	7.70 (3.18–18.63)	0.33 (0.17–0.65)

Data are from 75 critically ill children. AUC, area under the receiver-operating-characteristic curve; CI, confidence interval; LR+, positive likelihood ratio; LR−, negative likelihood ratio. LR+ = sensitivity/(1 − specificity); LR− = (1 − sensitivity)/specificity. Optimal thresholds were determined by Youden’s J statistic. Operating characteristics were not reported for sIL-2Rα in the mortality analysis because the AUC was below 0.50. ΔAUC values are computed from unrounded AUC estimates. ^a^ Combined logistic-regression model including log_10_ ferritin and sIL-2Rα (Hosmer–Lemeshow χ^2^ = 4.14, df = 6, *p* = 0.658; 5-fold pooled out-of-fold cross-validated AUC = 0.914). ^b^ Predicted-probability threshold. ^c^ Post hoc reduced mortality model retaining PRISM III and log_10_-transformed ferritin. The extended model additionally included sIL-2Rα, giving three candidate predictor parameters for 20 deaths; sIL-2Rα was not associated with mortality and did not improve the reported internal-validation performance. ^d^ Apparent AUC; bootstrap optimism-corrected AUC = 0.800. Model-specific cross-validation is reported in Supplementary Table S8, and paired added-performance analyses in Supplementary Table S11. Sensitivity and specificity 95% CIs were calculated using the Wilson method; likelihood-ratio 95% CIs were calculated.

**Table 4:** Multivariable logistic-regression models for HLH diagnosis and PICU mortality.

Variable	β (SE)	OR (95% CI)	*p* Value
**A|HLH diagnosis model**			
log_10_ (ferritin)	4.21 (1.04)	67.31 (11.22–1018.18)	**<0.001**
sIL-2Rα (ng/mL)	0.085 (0.030)	1.089 (1.029–1.169)	**0.002**
**B|PICU mortality model (parsimonious)**			
log_10_ (ferritin)	1.71 (0.51)	5.51 (2.18–17.24)	**<0.001**
PRISM III score	0.085 (0.037)	1.089 (1.014–1.180)	**0.019**

β, regression coefficient; CI, confidence interval; OR, odds ratio; PRISM III, Pediatric Risk of Mortality III; SE, standard error. Bold *p* values indicate statistical significance (*p* < 0.05). The HLH-diagnosis model (Panel A) was fitted with log_10_-transformed ferritin and untransformed sIL-2Rα; the mortality model (Panel B) is the post hoc reduced model retaining PRISM III and log_10_-transformed ferritin. The PRISM III-adjusted mortality analysis included 20 deaths and three candidate predictor parameters; the extended model additionally included sIL-2Rα, whose bootstrap coefficient interval included zero. Its inclusion did not improve the reported internal-validation performance (Supplementary Tables S3, S8 and S12). For log_10_-transformed ferritin, the odds ratio represents the change in odds per 10-fold increase in ferritin concentration. The very wide CI for ferritin in the HLH model reflects the strength of the ferritin–HLH association in a small sample; the diagnostic claim rests on discrimination (AUC, Table 3) rather than this odds-ratio magnitude. sIL-2Rα was modeled untransformed because, unlike ferritin, its distribution was approximately normal. *p* values are from profile penalized likelihood-ratio tests in the Firth models. In Panel B, the ferritin *p* value tests the addition of ferritin to the PRISM III-only model; the exact *p* value was 1.5 × 10^−4^ (Supplementary Table S11).

**Table 5:** Comparison of clinical and laboratory parameters by survival status.

Parameter	Non-Survivors (*n* = 20)	Survivors (*n* = 55)	*p* Value
Age, months	61 (11.8–130.2)	49 (11.5–110.0)	0.598
PRISM III score	22.5 (18.0–32.2)	18.0 (12.5–22.0)	**0.003**
PELOD score	31.5 (20.2–33.5)	21.0 (13.0–22.0)	**0.008**
PICU stay, days	13.5 (4.0–23.0)	10.0 (5.0–35.5)	0.461
Hospital stay, days	16.0 (12.8–33.0)	30.0 (14.0–57.0)	0.162
Ferritin, ng/mL	4730.5 (1190.2–38,947.8)	1007 (709.5–2000.0)	**<0.001**
sIL-2Rα, ng/mL ^†^	74.44 ± 19.89	73.99 ± 14.58	0.928
WBC, ×10^3^/μL	12.92 (5.19–19.01)	10.35 (5.81–14.92)	0.615
Hemoglobin, g/dL ^†^	10.22 ± 2.08	10.10 ± 2.46	0.830
Platelets, ×10^3^/μL	181.5 (47.2–317.2)	152 (88.5–329.0)	0.797
AST, U/L	51.5 (38.8–134.2)	49.0 (31.0–103.5)	0.628
ALT, U/L	34.0 (18.8–67.5)	31.0 (13.5–109.5)	0.569
Urea, mg/dL	35.5 (21.5–66.2)	23.0 (13.6–30.6)	**0.018**
Creatinine, mg/dL	0.50 (0.20–1.00)	0.30 (0.20–0.50)	0.155
Albumin, g/dL	3.0 (2.6–3.8)	3.3 (2.8–3.8)	0.425
CRP, mg/L	48.0 (8.1–133.8)	16.0 (3.3–64.0)	0.100
Procalcitonin, ng/mL	3.60 (0.70–20.8)	0.30 (0.10–6.80)	0.071
LDH, U/L	568.5 (341.8–1036.5)	472.0 (285.0–669.5)	0.263
INR	1.27 (1.12–1.61)	1.25 (1.08–1.49)	0.468
aPTT, s	32.5 (29.0–37.0)	29.0 (25.4–34.0)	**0.025**
Lactate, mmol/L	2.50 (1.70–3.80)	1.80 (1.30–2.90)	0.072

Data are median (IQR) unless otherwise indicated. Bold *p* values indicate statistical significance (*p* < 0.05); *p* values were calculated using the Mann–Whitney U test unless indicated. ^†^ Mean ± SD; compared using Student’s *t*-test (both groups passed the Shapiro–Wilk normality test, *p* > 0.05). PELOD, Pediatric Logistic Organ Dysfunction; PRISM III, Pediatric Risk of Mortality III. Other abbreviations as in Table 2.

## Data Availability

The raw data supporting the conclusions of this article will be made available by the authors, without undue reservation. Inquiries can be directed to the corresponding author.

## Supplementary Materials

The following supporting information can be downloaded at https://www.mdpi.com/article/10.3390/diagnostics16162600/s1, Supplementary Table S1. Subgroup correlation analysis of ferritin and sIL-2Rα with laboratory parameters; Supplementary Table S2. Multivariable logistic regression for mortality prediction (biomarker-only model); Supplementary Table S3. Extended mortality prediction model including PRISM III score; Supplementary Table S4. Model comparison statistics; Supplementary Table S5. Classification performance at optimal cutoffs; Supplementary Table S6. Disease composition of the “Other Diseases” group; Supplementary Table S7. Effect sizes (Cohen’s d) for between-group comparisons; Supplementary Table S8. Internal validation using cross-validation; Supplementary Table S9. Calibration of selected models; Supplementary Table S10. Paired comparison of standalone ferritin and sIL-2Rα AUCs; Supplementary Table S11. Complementary analyses of added prognostic information beyond PRISM III; Supplementary Table S12. Comparative performance of diagnostic and prognostic models; Supplementary Figure S1. Correlation heatmap of ferritin and sIL-2Rα with clinical parameters; Supplementary Figure S2. Ferritin and sIL-2Rα levels by diagnostic group; Supplementary Methods.
