# Supplementary material for: Diagnostic and Prognostic Value of Ferritin and Soluble Interleukin-2 Receptor α in Critically Ill Children with Hyperferritinemia
Source: Diagnostics (Basel). 2026 Aug 17;16(16):2600. doi: 10.3390/diagnostics16162600 (PMC13511872; doi:10.3390/diagnostics16162600)
Supplement: Supplementary file 1 [file diagnostics-16-02600-s001.zip › diagnostics-4405201-supplementary.pdf]

Supplementary Material

Diagnostic and Prognostic Value of Ferritin and Soluble Interleukin-2 Receptor α in Critically Ill Children with Hyperferritinemia

Contents

Supplementary Table S1. Subgroup correlation analysis of ferritin and sIL-2Rα with laboratory parameters

Supplementary Table S2. Multivariable logistic regression for mortality prediction (biomarker-only model)

Supplementary Table S3. Extended mortality prediction model including PRISM III score

Supplementary Table S4. Model comparison statistics

Supplementary Table S5. Classification performance at optimal cutoffs

Supplementary Table S6. Disease composition of the "Other Diseases" group

Supplementary Table S7. Effect sizes (Cohen's d) for between-group comparisons

Supplementary Table S8. Internal validation using cross-validation

Supplementary Table S9. Calibration of selected models

Supplementary Table S10. Paired comparison of standalone ferritin and sIL-2Rα AUCs

Supplementary Table S11. Complementary analyses of added prognostic information beyond PRISM III

Supplementary Table S12. Comparative performance of diagnostic and prognostic models

Supplementary Figure S1. Correlation heatmap of ferritin and sIL-2Rα with clinical parameters

Supplementary Figure S2. Ferritin and sIL-2Rα levels by diagnostic group

Supplementary Methods

**Supplementary Table S1 | ** Subgroup correlation analysis of ferritin and sIL-2Rα with laboratory parameters.

A | Ferritin correlations

Parameter	HLH (n = 25)	Sepsis (n = 25)	Other (n = 25)	P[†]	P[‡]
AST	0.273 (−0.136 to 0.603)	0.199 (−0.213 to 0.551)	0.134 (−0.276 to 0.503)	0.629	0.825
ALT	0.333 (−0.072 to 0.643)	0.190 (−0.222 to 0.544)	0.164 (−0.247 to 0.525)	0.550	0.930
LDH	0.219 (−0.193 to 0.565)	0.142 (−0.268 to 0.509)	−0.120 (−0.492 to 0.289)	0.256	0.382

B | sIL-2Rα correlations

Parameter	HLH (n = 25)	Sepsis (n = 25)	Other (n = 25)	P[†]	P[‡]
Triglyceride	0.314 (−0.093 to 0.631)	−0.009 (−0.403 to 0.387)	0.220 (−0.192 to 0.566)	0.737	0.441
ALT	0.196 (−0.216 to 0.549)	−0.220 (−0.566 to 0.192)	0.040 (−0.361 to 0.428)	0.598	0.383
INR	−0.132 (−0.501 to 0.278)	−0.158 (−0.520 to 0.253)	−0.346 (−0.652 to 0.057)	0.449	0.504

Data are Spearman ϱ (95% CI). [†]HLH vs other diseases (Fisher's Z). [‡]Sepsis vs other diseases (Fisher's Z); confidence intervals were calculated using Fisher's z transformation. ALT, alanine aminotransferase; AST, aspartate aminotransferase; HLH, hemophagocytic lymphohistiocytosis; INR, international normalized ratio; LDH, lactate dehydrogenase.

**Supplementary Table S2 | ** Multivariable logistic regression for mortality prediction (biomarker-only model).

Variable	β	SE	OR	95% CI	P value
$\log_{10}$(ferritin)	1.79	0.49	6.01	2.47–18.04	**<0.001**
sIL-2Rα (ng/mL)	−0.004	0.018	0.996	0.959–1.033	0.81

β, regression coefficient; CI, confidence interval; OR, odds ratio; SE, standard error. The Hosmer–Lemeshow test was nonsignificant (χ^2 = 6.98, df = 6, P = 0.32). sIL-2Rα was not associated with mortality in this model. At the optimal cutoff (0.55): sensitivity 45.0% (25.8–65.8), specificity 98.2% (90.4–99.7), LR+ 24.75 (3.34–183.18), LR− 0.56 (0.38–0.83); AUC 0.753 (95% CI 0.614–0.880).

**Supplementary Table S3 | ** Extended mortality prediction model including PRISM III score (three-predictor model).

Variable	β (SE)	OR (95% CI)	P	Bootstrap 95% CI for β
$\log_{10}$(ferritin)	1.69 (0.50)	5.43 (2.15–17.24)	**<0.001**	0.950 to 3.470
sIL-2Rα (ng/mL)	−0.010 (0.020)	0.990 (0.950–1.031)	0.62	−0.077 to 0.050*
PRISM III score	0.085 (0.037)	1.089 (1.014–1.181)	**0.019**	0.005 to 0.210

Model AUC 0.812; bootstrap optimism-corrected AUC 0.779; events per variable 6.7. At the optimal cutoff (0.32): sensitivity 70.0%, specificity 87.3%, LR+ 5.50, LR− 0.34. *The bootstrap interval for sIL-2Rα included zero, indicating substantial coefficient uncertainty. CI, confidence interval; EPV, events per variable; OR, odds ratio; PRISM III, Pediatric Risk of Mortality III; SE, standard error.

Supplementary Table S4 | Model comparison statistics.

Comparison	ΔAUC	95% CI	P value
**HLH diagnosis**			
Combined vs ferritin alone	+0.034	−0.119 to 0.187	0.663
Combined vs sIL-2Rα alone	+0.242	0.035 to 0.449	**0.022**
**Mortality prediction**			
Biomarker-only vs ferritin alone	−0.001	−0.098 to 0.096	0.992
Extended† vs biomarker-only	+0.059	−0.021 to 0.139	0.148
Parsimonious (ferritin + PRISM III) vs PRISM III alone	+0.087	−0.018 to 0.192	0.105

ΔAUC, difference in area under the curve; CI, confidence interval. P values are from DeLong's test for correlated ROC curves using apparent predictions from models fitted in the full cohort. †The extended model includes ferritin, sIL-2Rα, and PRISM III. The combined diagnostic model and the biomarker-only mortality model include $\log_{10}$-transformed ferritin and sIL-2Rα.

Supplementary Table S5 | Classification performance at optimal cutoffs (confusion matrices).

A | HLH diagnosis (combined model, probability cutoff = 0.34)

	Predicted non-HLH	Predicted HLH
Actual non-HLH (n = 50)	42	8
Actual HLH (n = 25)	3	22

B | Mortality prediction (biomarker-only model, probability cutoff = 0.55)

	Predicted survived	Predicted deceased
Actual survived (n = 55)	54	1
Actual deceased (n = 20)	11	9

C | Mortality prediction (parsimonious ferritin–PRISM III model, probability cutoff = 0.33)

	Predicted survived	Predicted deceased
Actual survived (n = 55)	50	5
Actual deceased (n = 20)	6	14

Optimal probability cutoffs were determined using Youden's J statistic. Overall accuracy: HLH diagnosis 85.3% (64/75); mortality prediction 84.0% (63/75); parsimonious ferritin–PRISM III model 85.3% (64/75).

Supplementary Table S6 | Disease composition of the "Other Diseases" group (n = 25).

Underlying condition	n	% of group
Malignancy (hematologic or solid tumors)	7	28.0
Cardiovascular disorders	4	16.0
Metabolic diseases	4	16.0
Bone marrow or solid-organ transplantation	2	8.0

Cerebral palsy (with or without comorbidities)	2	8.0
Other neurological sequelae[†]	2	8.0
Post-operative critical illness	1	4.0
Primary immunodeficiencies	1	4.0
Post-traumatic critical illness	1	4.0
Epilepsy	1	4.0

[†]Other neurological sequelae includes post-meningitis sequelae and similar conditions. Categories are mutually exclusive except where comorbidities are indicated. Patients were classified by the primary underlying condition necessitating PICU admission.

Supplementary Table S7 | Effect sizes (Cohen's d) for between-group comparisons.

A | Diagnostic-group comparisons

Variable	HLH vs Sepsis	HLH vs Other	Sepsis vs Other
Ferritin (log_{10})	1.16 (0.56–1.75)*	1.09 (0.50–1.68)*	−0.26 (−0.82 to 0.29)
sIL-2Rα	0.76 (0.19–1.34)†	0.80 (0.23–1.38)*	0.26 (−0.30 to 0.82)
AST (log_{10})	0.82 (0.24–1.39)*	0.41 (−0.15 to 0.97)	−0.37 (−0.93 to 0.19)
ALT (log_{10})	0.80 (0.23–1.38)*	0.09 (−0.47 to 0.64)	−0.46 (−1.02 to 0.10)
LDH (log_{10})	1.29 (0.68–1.90)‡	1.24 (0.63–1.84)‡	−0.01 (−0.56 to 0.55)
CRP (log_{10})	−0.33 (−0.89 to 0.23)	0.86 (0.28–1.43)*	1.11 (0.51–1.70)*
Albumin	−1.15 (−1.75 to −0.55)*	−1.72 (−2.37 to −1.07)‡	−0.49 (−1.05 to 0.07)
Calcium	−1.02 (−1.61 to −0.44)*	−0.28 (−0.84 to 0.28)	0.63 (0.07–1.20)†
aPTT	0.70 (0.13–1.27)†	0.88 (0.30–1.46)*	0.28 (−0.27 to 0.84)

B | Mortality comparison (non-survivors vs survivors)

Variable	Cohen's d (95% CI)	Interpretation
PRISM III score	0.95 (0.42–1.49)	Large*
PELOD score	1.67 (1.09–2.25)	Very large‡
Ferritin (log_{10})	0.80 (0.27–1.33)	Large*
Procalcitonin (log_{10})	0.58 (0.06–1.10)	Small to medium†

Data are Cohen's d (95% CI). Positive d indicates higher values in the first group (HLH or non-survivors). Effect-size interpretation: $|d| < 0.20$, negligible; †$0.20 \le |d| < 0.80$, small to medium; *$0.80 \le |d| \le 1.20$, large; ‡$|d| > 1.20$, very large. Log_{10} transformation was applied to skewed variables (ferritin, AST, ALT, LDH, CRP, procalcitonin) before effect-size calculation. PELOD, Pediatric Logistic Organ Dysfunction; PRISM III, Pediatric Risk of Mortality III.

Supplementary Table S8 | Internal validation using cross-validation.

A | Summary

Model	Apparent AUC	5-fold CV pooled OOF AUC† (min–max across 50 repetitions)	LOOCV	Apparent–OOF difference
HLH diagnostic: log_{10}(ferritin) + sIL-2Rα	0.933	0.914 (0.899–0.922)	0.908	0.019

Mortality, biomarker-only: $\log_{10}$(ferritin) + sIL-2Rα	0.753	0.704 (0.672–0.735)	0.683	0.049
Extended mortality: $\log_{10}$(ferritin) + sIL-2Rα + PRISM III	0.812	0.766 (0.736–0.798)	0.767	0.046
Parsimonious mortality: $\log_{10}$(ferritin) + PRISM III	0.811	0.781 (0.754–0.805)	0.776	0.030

AUC, area under the receiver-operating-characteristic curve; CV, cross-validation; LOOCV, leave-one-out cross-validation; OOF, out-of-fold; SD, sample standard deviation across folds. All models were estimated by Firth's penalized maximum likelihood and refitted within each fold. †The 5-fold CV estimate is the pooled OOF AUC averaged across 50 repetitions; the values in parentheses are the minimum and maximum across repetitions. Fold-specific cross-validation results are descriptive and not used for inference. Apparent–OOF difference = apparent AUC − pooled OOF AUC.

Supplementary Table S9 | Calibration of selected models (apparent and bootstrap-corrected).

Model	Brier (apparent → corrected)	Calibration slope (apparent → corrected)	Calibration intercept	Hosmer–Lemeshow χ^2 (df = 6)	P
HLH diagnosis	0.096 → 0.110	1.14 → 1.00	−0.02	4.14	0.658
Mortality (parsimonious)	0.126 → 0.138	1.08 → 1.02	−0.03	9.10	0.168

Brier score (lower = better), calibration slope, and calibration intercept were estimated from Firth-penalized predicted probabilities; corrected values are optimism-adjusted using 1,000 bootstrap resamples. The Hosmer–Lemeshow test used 8 groups (df = 6).

Supplementary Table S10 | Paired comparison of standalone ferritin and sIL-2Rα AUCs.

Outcome	Ferritin AUC	sIL-2Rα AUC	ΔAUC (95% CI)	P	Holm-adjusted P
Adjudicated HLH	0.898	0.690	0.208 (0.036–0.379)	0.018	0.019
PICU mortality	0.754	0.495	0.260 (0.064–0.455)	0.009	0.019

AUC, area under the receiver-operating-characteristic curve. Individual AUC 95% CIs are reported in main-text Table 3. Because both biomarkers were measured in the same participants, AUC differences were estimated using paired DeLong tests. These two targeted post hoc comparisons were treated as one family and adjusted using Holm's method. ΔAUC values and Holm-adjusted P values are computed from unrounded estimates. The diagnostic comparison remains conditioned on a ferritin-informed reference process and represents greater observed discrimination within this cohort rather than assay-independent superiority.

Supplementary Table S11 | Complementary analyses of added prognostic information beyond PRISM III.

Added predictor	DeLong ΔAUC (95% CI)	DeLong P	Bootstrap ΔAUC (2.5th–97.5th percentile)	Repeated-CV ΔAUC (2.5th–97.5th percentile)	Penalized LRT P
Ferritin (log_{10})	0.087 (−0.018 to 0.192)	0.105	0.087 (0.005 to 0.189)	0.083 (0.055 to 0.114)	1.5×10^{-4}
sIL-2Rα	−0.001 (−0.024 to 0.022)	0.938	0.005 (−0.029 to 0.086)	−0.027 (−0.074 to 0.008)	0.826

Each row compares a two-predictor Firth model containing PRISM III and the added predictor with the PRISM III-only model. DeLong and bootstrap analyses quantify changes in AUC; the paired bootstrap used 1,000 resamples with both models refitted in each resample, and percentile intervals were obtained from the empirical ΔAUC distribution. Repeated 5-fold cross-validation used 200 repetitions with identical folds for the paired models; the reported interval is the 2.5th–97.5th percentile across repetitions and is not a confidence interval. The penalized likelihood-ratio P value tests the added predictor; for ferritin, this corresponds to the ferritin coefficient test reported in main-text Table 4, Panel B. These analyses were performed post hoc. ΔAUC values are computed from unrounded AUC estimates.

Supplementary Table S12 | Comparative performance of diagnostic and prognostic models.

Model	EPP	Apparent AUC	Optimism-corrected AUC	Bootstrap optimism-corrected Brier score	Slope
**Diagnostic (outcome: adjudicated HLH)**					
Ferritin (log_{10})	25.0	0.898	0.896	0.128	1.04
sIL-2Rα	25.0	0.690	0.687	0.209	1.96
Ferritin + sIL-2Rα	12.5	0.933	0.924	0.110	1.00
**Prognostic (outcome: PICU mortality)**					
PRISM III	20.0	0.725	0.723	0.183	1.20
PRISM III + ferritin	10.0	0.811	0.800	0.138	1.02
PRISM III + sIL-2Rα	10.0	0.724	0.691	0.188	0.94
PRISM III + ferritin + sIL-2Rα	6.7	0.812	0.779	0.141	0.93

EPP, events per predictor parameter. All models were fitted using Firth's penalized maximum likelihood. Optimism-corrected AUCs, Brier scores, and calibration slopes were obtained using 1,000 bootstrap resamples with each model refitted in every resample. The three-predictor prognostic model had 6.7 events per parameter and is shown for completeness; the two-predictor ferritin–PRISM III model was retained as the parsimonious main-text model.

Supplementary Figures

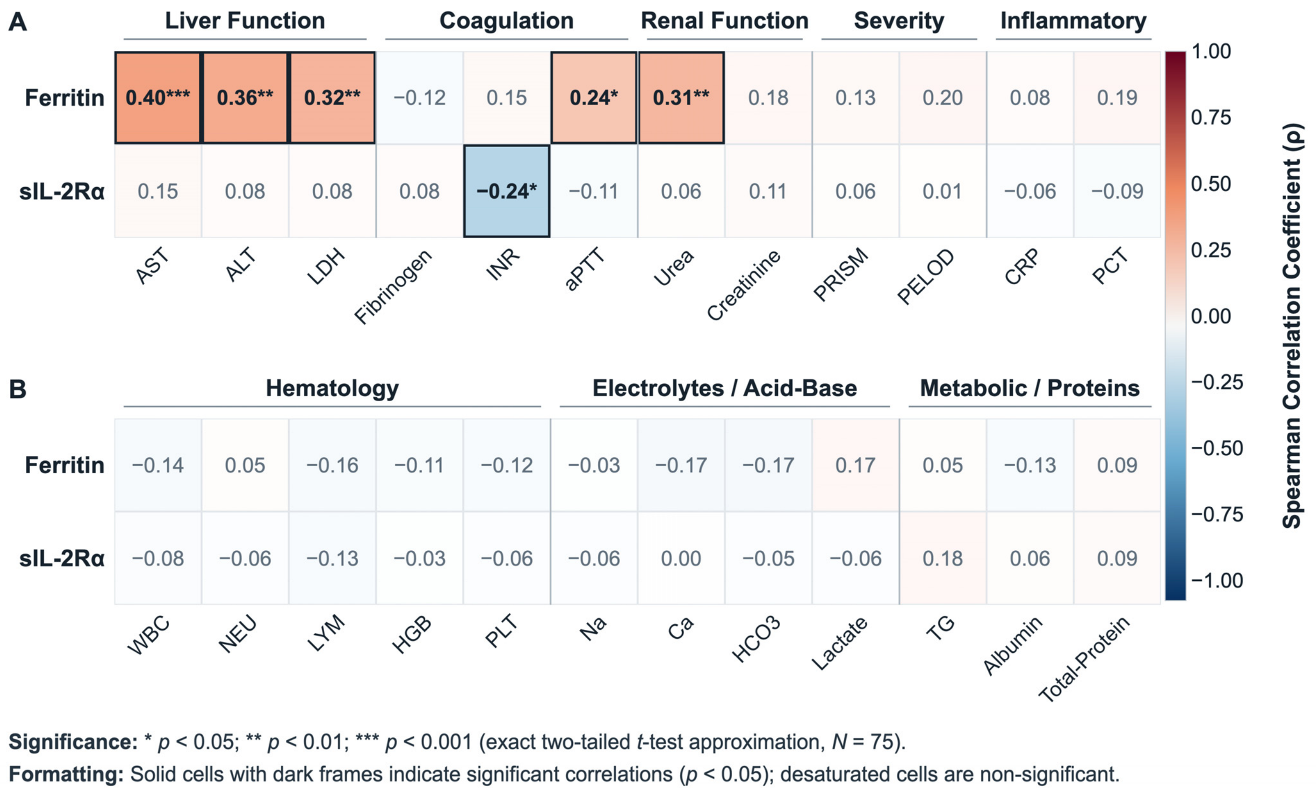

Supplementary Figure S1 | Correlation heatmap of ferritin and sIL-2Rα with clinical and laboratory parameters across the entire cohort (n = 75). (A) Liver function, coagulation, renal function, severity and inflammatory markers; (B) hematology, electrolyte and acid–base, and metabolic and protein parameters. The color scale represents Spearman's ϱ. Asterisks denote unadjusted two-sided P values across 48 secondary tests. No multiplicity correction was applied; these correlations are exploratory.

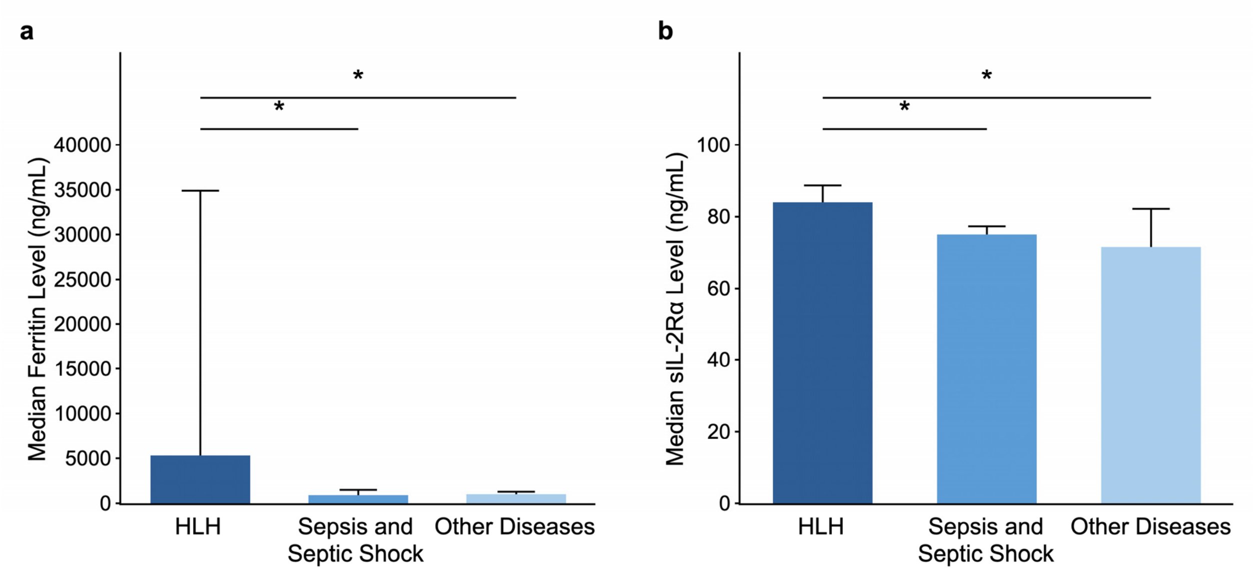

Supplementary Figure S2 | Ferritin (a) and sIL-2Rα (b) levels by diagnostic group (n = 25 per group). Bars show the median, and the upper whisker extends from the median to the third quartile. Groups were compared using the Kruskal–Wallis test followed by Dunn's pairwise tests with Benjamini–Hochberg adjustment. Ferritin was higher in HLH than in sepsis/septic shock and other diseases (both P < 0.001). sIL-2Rα was higher in HLH than in sepsis/septic shock (P = 0.029) and other diseases (P = 0.015). The two non-HLH groups did not differ for ferritin (P = 0.80) or sIL-2Rα (P = 0.861). *P < 0.05 for the bracketed comparison.

Supplementary Methods

Power Analysis

The required sample size was calculated a priori using G*Power software (version 3.1.9.7) for an omnibus one-way ANOVA comparing three independent groups. Based on prior literature reporting large between-group differences in ferritin and sIL-2Rα levels, a large effect size (Cohen's f = 0.40) was assumed. With α = 0.05 and 80% power, the minimum total sample size was 66 patients (22 per group). The planned sample size was 75 patients (25 per group), corresponding to 86.9% power under the assumed effect size.

Bootstrap Validation

Bootstrap validation was performed with 1,000 resamples to quantify model optimism and coefficient uncertainty. In each resample, the model was refitted and evaluated in both the bootstrap sample and the original dataset. The optimism-corrected AUC was calculated as the original AUC minus mean optimism. Percentile intervals for regression coefficients were derived from the bootstrap distributions.

Cross-Validation

Stratified 5-fold cross-validation was performed to assess internal model stability. The dataset was divided into five folds preserving outcome proportions; each model was refitted in four folds and evaluated in the held-out fold. The model-level analysis in Supplementary Table S8 used 50 repetitions and additionally reported leave-one-out cross-validation. The targeted paired ΔAUC analysis used 200 repeated 5-fold partitions (Supplementary Table S11). The apparent–OOF difference was calculated as the apparent AUC minus the pooled out-of-fold AUC. These procedures provide internal validation and do not establish external validity.

Model Specification and Internal Validation

Predictor number was limited to reduce overfitting given 20 mortality events. The three-predictor mortality model had 6.7 events per parameter and is reported for completeness; the post hoc two-predictor model excluding sIL-2Rα had 10 events per parameter and was retained as the parsimonious main-text model. Model performance was assessed using bootstrap validation, repeated stratified 5-fold cross-validation, and leave-one-out cross-validation, as detailed above.

Additional Post Hoc Analyses

Standalone ferritin and sIL-2Rα AUCs were compared for adjudicated HLH and PICU mortality using paired DeLong tests because both biomarkers were measured in the same participants. These two targeted post hoc comparisons were treated as one family and adjusted using Holm's method (Supplementary Table S10). For prognostic analyses, PRISM III plus ferritin and PRISM III plus sIL-2Rα were each compared with PRISM III alone using apparent paired DeLong ΔAUC, a paired bootstrap distribution of ΔAUC, repeated cross-validated ΔAUC, and Firth penalized likelihood-ratio tests. The paired bootstrap distribution used 1,000 resamples with both models refitted in each resample; percentile intervals were obtained from the empirical ΔAUC distribution. Stratified 5-fold cross-validation was repeated 200 times using identical folds for the paired models; the mean out-of-fold ΔAUC and the 2.5th–97.5th percentile across repetitions were reported. Model-specific cross-validation estimates are reported in Supplementary Table S8, whereas paired added-performance comparisons are

reported in Supplementary Table S11. Comparative apparent and optimism-corrected performance across diagnostic and prognostic models is summarized in Supplementary Table S12.

Multiplicity. Benjamini–Hochberg adjustment was applied to Dunn's pairwise tests after significant Kruskal–Wallis tests, and Holm adjustment was applied to the two targeted paired standalone AUC comparisons. No further multiplicity correction was applied to secondary laboratory, correlation, or survival-status comparisons; these results are interpreted as exploratory. All 75 enrolled participants had complete data for ferritin, sIL-2Rα, PRISM III, PELOD, and PICU vital status; no imputation was used in the primary diagnostic or prognostic analyses.

Model Equations

HLH diagnostic model: $\text{logit}(P) = -21.30 + 4.21 \times \log_{10}(\text{ferritin}) + 0.085 \times \text{sIL-2R}\alpha$

Mortality model (biomarker-only): $\text{logit}(P) = -6.77 + 1.79 \times \log_{10}(\text{ferritin}) - 0.004 \times \text{sIL-2R}\alpha$

Mortality model (parsimonious, main-text model): $\text{logit}(P) = -8.61 + 1.71 \times \log_{10}(\text{ferritin}) + 0.085 \times \text{PRISM III}$

Probability is calculated as $P = 1 / [1 + \exp(-\text{logit})]$.
